# Building a drug ontology based on RxNorm and other sources

**DOI:** 10.1186/2041-1480-4-44

**Published:** 2013-12-18

**Authors:** Josh Hanna, Eric Joseph, Mathias Brochhausen, William R Hogan

**Affiliations:** 1 Division of Biomedical Informatics, University of Akransas for Medical Sciences, Little Rock, AR, USA

**Keywords:** Drug Ontology, RxNorm, ChEBI

## Abstract

**Background:**

We built the Drug Ontology (DrOn) because we required correct and consistent drug information in a format for use in semantic web applications, and no existing resource met this requirement or could be altered to meet it. One of the obstacles we faced when creating DrOn was the difficulty in reusing drug information from existing sources. The primary external source we have used at this stage in DrOn’s development is RxNorm, a standard drug terminology curated by the National Library of Medicine (NLM). To build DrOn, we (1) mined data from historical releases of RxNorm and (2) mapped many RxNorm entities to Chemical Entities of Biological Interest (ChEBI) classes, pulling relevant information from ChEBI while doing so.

**Results:**

We built DrOn in a modular fashion to facilitate simpler extension and development of the ontology and to allow reasoning and construction to scale. Classes derived from each source are serialized in separate modules. For example, the classes in DrOn that are programmatically derived from RxNorm are stored in a separate module and subsumed by classes in a manually-curated, realist, upper-level module of DrOn with terms such as 'clinical drug role’, 'tablet’, 'capsule’, etc.

**Conclusions:**

DrOn is a modular, extensible ontology of drug products, their ingredients, and their biological activity that avoids many of the fundamental flaws found in other, similar artifacts and meets the requirements of our comparative-effectiveness research use-case.

## Background

Several researchers have identified use cases for an ontology of drugs, such as comparative effectiveness research [1], clinical decision support [2-4], and clinical data warehousing and data integration [2,3,5-7], among others. We previously analyzed existing terminology and ontology artifacts that represent some aspect of drugs, and found that no existing resource was sufficient for our use cases in these domains [8]. Our requirements included (1) a historically comprehensive list of NDCs, (2) correctness with respect to pharmacology and biomedical science, (3) logically consistent, correct axioms that do not entail untrue or inconsistent inferences, and (4) interoperability with other ontologies used in translational science. Specifically, we analyzed RxNorm, the National Drug File – Reference Terminology, SNOMED CT, Chemical Entities of Biological Interest (ChEBI), an OWL conversion of the Anatomical and Therapeutic Chemical classification system, DrugBank, PharmGKB, and other sources and found that none of them met these requirements. Minimally, no existing resource contains in its current version a historically comprehensive list of National Drug Codes (NDCs). We also found problems with scientific correctness and unintended and incorrect description logic entailments from artifacts represented in Web Ontology Language. A key flaw inherent in several artifacts that frequently led to scientifically incorrect representations of drugs was assigning the properties of drug products such as tablets, creams, ointments, etc. to molecules and vice versa.

We therefore decided to build the Drug Ontology (DrOn) to meet our requirements. However, rather than assemble comprehensive historical information about drug products from scratch, we decided to begin with resources that (1) had enough quality information about drugs to begin with, (2) could therefore be restructured in a coherent manner, and (3) were sufficiently open to allow us to make DrOn publicly available.

RxNorm [5]—a standard drug terminology maintained by the U.S. National Library of Medicine (NLM)—includes normalized names and relationships extracted from several proprietary drug knowledge bases. Because RxNorm (1) contains a large amount of drug information, (2) is freely available, and (3) has a great deal of content available under a permissive license that allows derivative works, it is a good candidate for a source of information to create a formal drug ontology. In particular, RxNorm had enough information to allow us to rectify numerous shortcomings of existing artifacts, and its historical versions collectively contain the largest, openly available set of historical NDCs, dating back until June 2008 at least (the date of the first version of RxNorm to contain NDCs).

RxNorm is focused primarily on prescription and over-the-counter drugs that are currently available in the United States. It uses Concept Unique Identifiers called RXCUIs to catalog and relate information.

At this stage of DrOn development, we are interested in the ability to query for historical NDCs to study pharmacy claims databases that contain a decade or more of data (and thus NDCs in use 10 years ago as well as today are required). The NDC is a unique identifier that the Drug Listing Act of 1972 requires companies to report to the Food and Drug Administration (FDA). RxNorm associates each NDC with a drug product via the RXCUI. Although our requirement is to have a comprehensive, historical list of NDCs, RxNorm maintains only currently active NDCs in its current release. So tracking all NDCs and the RXCUIs with which they have been associated over historical releases of RxNorm is key to building DrOn, and represents a key contribution of the present work.

Moreover, NDCs are often lost with no explanation when an RXCUI is retired, especially in releases of RxNorm prior to 2009. This situation necessitates careful tracking to ensure that all valid NDCs (and, indeed, any useful information) associated with a retired RXCUI can be associated with the most recent RXCUI that refers to the same entity.

In this paper, we describe how we build DrOn from historical releases of RxNorm while navigating these pitfalls. In addition, during the build process, we map drug ingredients from RxNorm to the Chemical Entities of Biological Interest (ChEBI) ontology [9]. For example, we map the RXCUI for furosemide (4603) to the ChEBI identifier for furosemide: http://purl.obolibrary.org/obo/CHEBI_47426. As a result, we import hundreds of ChEBI classes and their associated URIs, labels, etc. into DrOn.

## Methods

The overall workflow of the extraction and translation process has three main steps:

1. Extracting relevant data from RxNorm, including information found only in older releases.

2. Transforming and loading this data into a normalized Relational Database Management System (RDBMS).

3. Translating the normalized RDBMS into an OWL 2.0 artifact.

Each of these three steps is further subdivided into substeps that we explain in detail below.

### Extracting information from RxNorm

We first download the raw RxNorm files directly from the NLM website, specifically the UMLS (or Unified Medical Language System) Terminology Services (UTS) site [10] and import them into a locally hosted RDBMS using the scripts provided by the NLM. Additionally, to support maintenance of comprehensive information over time, we created and maintain two additional tables that store all the information that we extract from each release of RxNorm (a subset of all the information). We describe these tables in detail below (sections entitled *Extraction of National Drug Codes (NDCs) and related RXCUIs* and *Tracking Provenance*).

Currently, we include in DrOn information from every version of RxNorm released between June, 2008 and February, 2013. The release from June, 2008 marks the first time that RxNorm-curated NDCs were included.

It should be noted that we use only information curated within RxNorm and not any information from its sources directly, and thus our overall process is allowable under the UMLS license (all content reused in DrOn is marked Level 0, which, per the license, does not prohibit derivative works as do levels 1 and higher).

#### RxNorm files

The next step is to extract all relevant information from the files downloaded from the UTS site. RxNorm comes as a set of nine Rich Release Format (RRF) files, each of which contains a specific subset of the total information. However, we process only five of these files in our build process.

Specifically, we process RXNSAT.RRF, RXNCONSO.RRF, RXNCUI.RRF, RXNCUICHANGES.RRF, and RXNSAB.RRF. Table 1 shows the information we extract from each file.

**Table 1:** The RxNorm files and the information extracted from each

**File**	**Extracted information**
RXNSAT.RRF	NDCs and RXCUIs
RXNCONSO.RRF	SCDFs, SCDs, SBDs, and INs and their RXCUIs
RXNCUI.RRF	retired RXCUIs with provenance
RXNCUICHANGES.RRF	RXCUI provenance
RXNSAB.RRF	RxNorm version information

There are four different “term types” in RXNCUI.RRF that are relevant to DrOn. They are: (1) Semantic Clinical Drug Forms (SCDFs), (2) Semantic Clinical Drugs (SCDs), (3) Semantic Branded Drugs (SBDs), and (4) Ingredients (IN). RxNorm treats NDCs as attributes of an SCD or SBD rather than a separate term type.

#### RXCUI provenance

**Figure 1:**
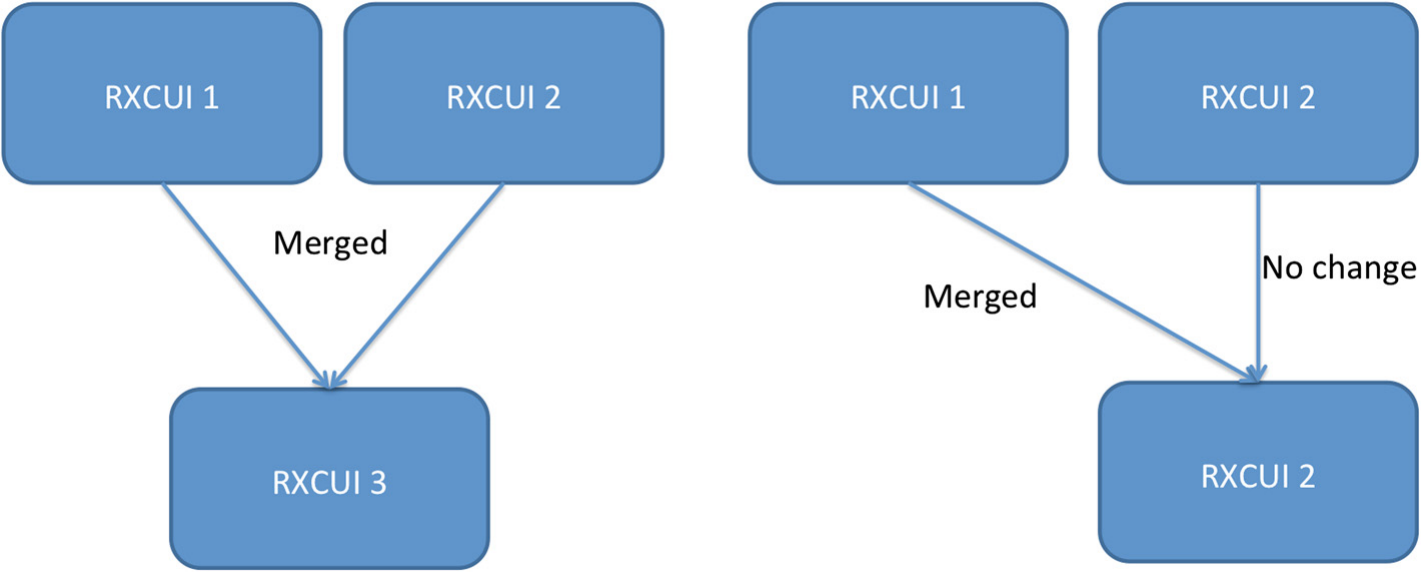
**How RXCUIs are de-duplicated.** How the National Library of Medicine handles RXCUI errors within RxNorm.

Tracking entities within RxNorm requires tracking the RXCUIs to which they are attached. This can be a difficult task. Any RXCUIs that have been entered in error are retired. Additionally, if two RXCUIs refer to the same entity, they are consolidated and either (1) one of them is retired while the other remains or (2) a new RXCUI is created and both older RXCUIs are retired (Figure 1). Prior to the April 2009 release of RxNorm, no comprehensive list of retired RXCUIs was included in RxNorm. The reasons for retirement are not always well-documented, making it difficult to distinguish between RXCUIs that have been retired because they are nonsense and ones that have been replaced or merged. For instance, as of this writing, there are 40 RXCUIs with 210 associated NDCs that are no longer contained in the most recent release of RxNorm, however, there is no record of why these RXCUIs were removed.

#### Extraction of National Drug Codes (NDCs) and related RXCUIs

To facilitate the tracking of NDCs, we have created an additional table, NDC_COMP, that contains a comprehensive list of all RxNorm-curated NDCs from all releases of RxNorm since June 2008 (when they first appeared) and their corresponding RXCUIs. To generate this table, we parse the RXNSAT.RRF file in each release of RxNorm. Any entry in the file whose source is RxNorm and is annotated as being an NDC is extracted from the file, along with its associated RXCUI, and imported into our NDC_COMP table. We also store the version from which each NDC was mined, which is parsed from the RXNSAB.RRF file.

#### Tracking provenance

The second of the two additional tables is a master conversion table, DEPRECATED_RXCUIS, which we use to track the current status of each retired RXCUI. This table contains two fields: old_rxcui and new_rxcui. The old_rxcui field contains a retired RXCUI, and the new_rxcui field contains the current RXCUI to which the retired RXCUI’s information is now associated. The new_rxcui field may also contain a status code if the retired RXCUI’s information is unable to be tracked because it was entered in error or split into multiple new RXCUIs. These special status codes are “ERROR” for RXCUIs that have been entered in error and “S_RXNCUI” for RXCUIs which have been split. Because RxNorm does not document why an erroneous RXCUI was entered in error, we are unable to do further processing on them or their associated information. For the RXCUIs which are split, it may be possible to track some of their associated information, but it is not always clear which information belongs to which child RXCUI and this issue requires manual intervention at present.

**Figure 2:**
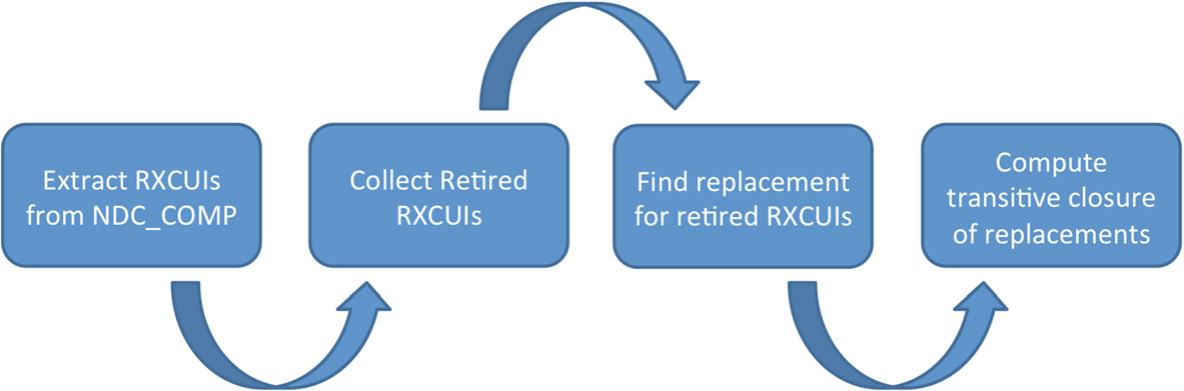
**Workflow for updating the DEPRECATED_RXCUIS table.** How we tracked RXCUI provenance.

Our DEPRECATED_RXCUIS table is updated with each release of RxNorm through the following procedure (Figure 2):

1. First, we extract any RXCUIs from the comprehensive NDC_COMP table, built as described above (section entitled *Extraction of National Drug Codes (NDCs) and related RXCUIs*), that can no longer be found in the RXNCONSO.RRF file being imported. We then import these RXCUIs into the old_rxcui column of our DEPRECATED_RXCUIS table. Because the RXNCONSO.RRF file contains all current RXCUIs, any RXCUIs that meet the above criteria must have been retired.

2. Next, using the RxNorm-curated RXNCUI table, we update all entries in the new_rxcui column. The RXNCUI table contains a *cui1* field containing a retired RXCUI, a *cui2* field containing the RXCUI into which the retired RXCUI’s information has been merged, and a cardinality column contains the number of RXCUIs into which the information has been merged. Any RXCUI that has been entered in error is indicated by an entry in which the value of the *cui1* field is equal to the value of the *cui2* field. Additionally, any entry with a cardinality greater than 1 indicates that the RXCUI has been split. These are indicated in our table by setting the new_rxcui entry to “ERROR” and “S_RXNCUI”, respectively. As of this writing, 768 RXCUIs and 3,484 associated NDCs are reported by RxNorm to have been entered in error and are therefore not included in DrOn. Additionally, 187 RXCUIs and 3,126 associated NDCs have been split. Both these RXCUIs and NDCs have also been left out of DrOn (for the time being) due to the difficulty of determining which information from the parent RXCUI belongs to which child RXCUI.

3. Finally, we compute the transitive closure, associating each RXCUI with the latest RXCUI that refers to the same entity with no intervening steps in our DEPRECATED_RXCUIS table. Because this table is updated with each release of RxNorm, occasionally an RXCUI in the new_rxcui field is retired. In such situations, the new_rxcui field is updated as described in Step 2, and a new row in the table is created with the newly-retired RXCUI set as the old_rxcui, and the new_rxcui field is set to match the updated new_rxcui from the original entry.

### Mapping to ChEBI

The process maps ingredients (IN entity type) extracted from RxNorm to ChEBI Uniform Resource Identifiers (URIs) where possible. We accomplish this step through a simple Java console application (that we built) that compares the labels of ingredients pulled from RxNorm with class annotations in ChEBI. We assumed that any exact matches between the names or synonyms of RxNorm IN entities and ChEBI annotations meant that the RxNorm concept and ChEBI class referred to the same entity, and thus we used the ChEBI URIs in DrOn for the ingredient. We used three different annotation types from ChEBI in the mapping process: rdfs:label, related_synonym, and exact_synonym. To date, we import into DrOn ~750 classes (including URI and rdfs:label and other annotations) from ChEBI: roughly 500 matches were on rdfs:label, 250 were on related_synonym, and only two were on exact_synonym. Many of the ingredients found in RxNorm are extracts of various plants, e.g. ginger extract, which we would not expect to find in ChEBI. These ingredients are currently all children of the class *processed material*, which we imported from the Ontology of Biomedical Investigations (OBI).

The process originally mapped somatropin (also known as somatotroin or human growth hormone) erroneously to the ChEBI role 'growth hormone’. Once we noticed this error, we fixed it. The ingredient is now mapped to the Protein Ontology URI that represents the protein molecule somatotropin.

We assigned a DrOn URI to every ingredient that was not found in ChEBI via this process.

### Transforming the data into a normalized format

**Figure 3:**
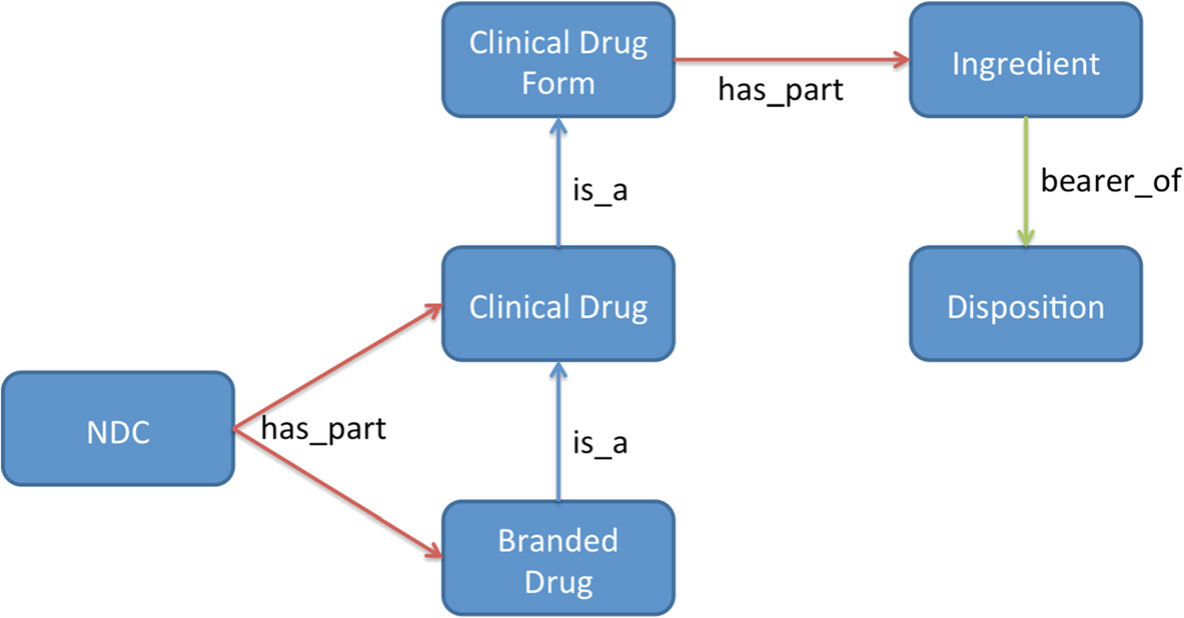
**DrOn Entity Types.** The entity types of DrOn and their relationships as stored in the normalized format.

As noted above, there are five RxNorm term types that we were initially interested in pulling from RxNorm, including ingredient, clinical drug form, clinical drug, branded drug, and national drug code (NDC). Additionally, we wanted to represent a number of dispositions of ingredients, such as the disposition of metoprolol to bind beta-adrenergic receptors of cells. Figure 3 shows these six entity types and the relationships between them. Note that the entities the NDC classes represent are not the codes themselves, but instead the packaged drug products that the NDCs represent. Additionally, every DrOn entity that corresponds to a RxNorm entity is annotated with the corresponding RXCUI via an annotation property called *has_Rxcui*.

#### Entity types

The **ingredient** entities represent the types of molecules that are present in a drug product and have an active biological role. The URIs of ingredients, where possible, are taken from the Chemical Entities of Biological Interest (ChEBI) ontology as described above. Examples of ingredients include acetaminophen, sulfur, and ephedrine*.* There are 7,848 unique ingredients in DrOn.

The **disposition** entities represent dispositions that molecules bear (see Hogan et al. [8]) that correspond to what is typically considered a drug’s mechanism of action. There are, as of now, six molecular dispositions in DrOn. They are:

1. *non-activating competitive beta-adrenergic receptor binding disposition* (i.e., beta-adrenergic blockade)

2. *function-inhibiting hydrogen/potassium adenosine tri-phosphatase enzyme (H+/K + ATPase) binding disposition* (i.e., proton-pump inhibition)

3. *function-inhibiting L-type voltage-gated calcium channel binding disposition* (i.e., the subtype of calcium-channel blockade found in cardiovacscular drugs that lower blood pressure and alter heart rhythm)

4. *function-inhibiting vitamin K epoxide reductase binding disposition* (i.e., the type of Vitamin K antagonism exhibited by warfarin)

5. *function-inhibiting Na-K-Cl cotransporter 2 (NKCC2) binding disposition* (i.e., NKCC2 inhibition)

6. *function-inhibiting T-type calcium channel binding disposition* (i.e., another subtype of calcium-channel blockade, which does not have cardiovascular effects)

These six dispositions were chosen based on their biological importance and relevance to ongoing comparative effectiveness research at the University of Arkansas for Medical Sciences. There is no direct correspondence between DrOn dispositions and RxNorm, because by design RxNorm lacks information about drugs’ mechanism of action. Instead, the relationships between DrOn dispositions and ingredients were mined from ChEBI, although ChEBI treats the same realizable entities that we represent as roles [8]. Table 2 shows the associated ChEBI role from which the ingredient relationships for the three dispositions were mined. Authors WRH and JH manually curated the other three dispositions not in the table.

**Table 2:** The ChEBI roles used to mine DrOn disposition-ingredient relationships

**DrOn disposition**	**ChEBI role**
non-activating competitive beta-adrenergic receptor binding disposition	beta-adrenergic antagonist
function-inhibiting hydrogen/potassium adenosine triphosphatase enzyme (H+/K + ATPase) binding disposition	proton pump inhibitor
function-inhibiting L-type voltage-gated calcium channel binding disposition	calcium channel blocker

*Function-inhibiting T-type calcium channel binding disposition* was included because we erroneously associated *ethosuximide* and *function-inhibiting L-type voltage-gated calcium channel binding disposition*. This error was not due to any particular oversight of ChEBI but an artifact caused by the more specific nature of DrOn’s dispositions as compared to ChEBI’s more general c*alcium channel blocker*. Ethosuximide instead is the bearer of a *function-inhibiting T-type voltage-gated calcium channel binding disposition*, which does not confer any cardiovascular activity but instead gives it a neurological, anti-seizure activity.

The **Clinical Drug Form** (CDF) entities represent types of drug products at the level of granularity of dose form (e.g. drug tablet) and often the intended route of administration (e.g. oral ingestion), without brand or strength information. They correspond with SCDFs in RxNorm. Examples of CDFs include *estradiol transdermal patch, iodine topical solution,* and *menthol crystals.* There are 14,035 unique CDFs in DrOn.

The **Clinical Drug** (CD) entities represent drug products at the level of granularity of specific dosage/strength/form information. They are related to the CDF by an is-a relationship. For example, every *aspirin 325 MG enteric coated tablet* (CD) is a *aspirin enteric coated tablet* (CDF). DrOn contains 34,560 CDs.

The **Branded Drug** (BD) entities represent brand-name drug products with specific dosage/strength/form information. The drug products that BDs represent are related to the products that CDs represent by an is-a relationship (RxNorm uses a “tradename of” relationship, but drug products are not names). There are 21,248 unique BDs in DrOn.

The **National Drug Code** (NDC) entities represent a drug product and its packaging, such as a 100 tablet bottle of acetaminophin 325 mg oral tablets. These entities are distinct from entities represented by BDs or CDs, instead containing some number of instances of drug products represented by CDs/BDs, for example a 100-tablet bottle of aspirin 325 mg tablets. There are 390,813 unique NDC entities in DrOn (Table 3).

**Table 3:** The associated RxNorm entity type for each DrOn entity except disposition

**DrOn entity type**	**RxNorm entity type**
CDF	SCDF
CD	SCD
BD	SBD
Ingredient	IN
NDC	SCD or SBD attribute

#### RDBMS design

The RDBMS design representing the normalized format of the entity types described above is simple. There are 5 core tables, one for each entity type. These are as follows: *clinical_drug_form, clinical_drug, branded_drug, ndc, ingredient,* and *disposition.*

Additionally, there are two tables storing provenance information from RxNorm, such as the version of RxNorm in which each RxCUI was found. These are *rxcui* and *rxnorm*. These are completely separate from the core entity tables to allow for incorporation of other data.

Many-to-many tables representing the relationships between the various entities are omitted in the interest of brevity. However, all of the relationships shown in 1 are also represented in RDBMS.

#### Export into RDBMS system

The export process is done in four major steps:

1. First, we initialize the rxcui and rxnorm tables. This includes mapping every deprecated RXCUI to the most recent RXCUI that identifies the same object, either to an RXCUI from the current set or another deprecated, but not entered in error, RXCUI.

2. Next, we initialize the ndc table. This primarily involves copying all the NDCs found in the extraction process (without the duplication caused by storing NDCs multiple times during the process) and associating them with the relevant RXCUI.

3. Next, we create the ingredients, CDFs, CDs, and BDs from the associated RxNorm type. This includes maintaining the proper relationships between the various entities (e.g. associating the correct ingredients with each CDF).

4. Finally, we associate each NDC with the appropriate CD or BD. This primarily involves following the provenance trail of RXCUIs provided in step.

### Creating the OWL 2.0 Artifact

We use the OWLAPI 3.4.3 [11], Scala 2.10 [12], and Slick 1.0.0 [13] to extract the entities from our internal representation and transform them into an OWL artifact. This process is subdivided into the following steps:

1. Extract the ingredients, using ChEBI URIs where appropriate.

2. Extract the dispositions and associate them via the *bearer_of* relation to the one or more ingredients.

3. Extract the clinical drug forms and associate them via the *has_proper_part* relation to the one or more ingredients.

4. Extract the clinical drugs and assert they are a subclass of the appropriate clinical drug form.

5. Extract the branded drugs and assert that they are a subclass of the appropriate clinical drug.

6. Extract the NDCs and assert that they are related to one branded drug or one clinical drug via the *has_proper_part* relation.

This ordering of the steps is deliberate. Each step depends on one or more previous steps.

Since the RDBMS structure defined above represents the entities and their relationships already, this process is fairly straightforward.

## Results and discussion

We developed an ontology, DrOn, that contains information programmatically derived from three different sources (RxNorm, ChEBI, and PRO) during its build process. Because it is derived from general-purpose resources, we believe DrOn can serve many use cases beyond our current ones (although this conjecture requires further research). We plan on adding additional sources in the future to maintain current information in DrOn, with more immediate plans to include information from Structured Product Labels [14]. As such, we built our internal representation to maintain provenance information of the sources separately, ensuring that we can both track the provenance of the various entities as the ontology develops and add new sources without adversely affecting the existing ontology.

DrOn follows OBO Foundry guidelines and is currently listed on the OBO Foundry website as a candidate ontology. In additional to the information from RxNorm detailed above, DrOn imports BFO 1.1 and includes terms MIREOTed from the Relationship Ontology, the Ontology of Biomedical Investigations, and BFO 2.

DrOn contains a total of 514,268 classes as of this writing. Of these, 2 are MIREOTed, 51 were imported using OWL’s built in mechanisms, 1,885 were taken from ChEBI, two were taken from PRO, and the remaining 512,328 were mined from RxNorm.

The development site and issue tracker for DrOn can be found at https://bitbucket.org/uamsdbmi/dron. The permanent URL for DrOn is http://purl.obolibrary.org/obo/dron.owl. DrOn can also be found in Ontobee [15]. It can be browsed and queried at http://www.ontobee.org/browser/index.php?o=DRON.

### The upper module of DrOn

DrOn is primarily made up of classes created by extracting information from other sources. However, there are a number of classes that were defined specifically for DrOn, which we use to give structure to the extracted data, as well as to handle future work such as representing dose forms.

We define **'drug product’** as 'a material entity (1) containing at least one scattered molecular aggregate as part (the active ingredient) and (2) that is the bearer of a clinical drug role’. It is currently the superclass to all CDFs (and thus, all CDs and BDs).

Additionally, we define in DrOn several different subtypes of drug product, such as '**drug capsule**’ and '**drug tablet**’. Currently, we do not use these subclasses, but we will eventually place all the drug products under them to achieve a better mid-level structure.

As stated above, we imported the '**processed material**’ class from the OBI. OBI defines it as 'a material entity that is created or changed during material processing’. It is the parent class of all the drug ingredients that we could not match to a ChEBI class (and thus are not found within the ChEBI structure). Given that most of these ingredients are plant extracts created by some form of processing a plant, it was the most appropriate choice for the time being.

The '**packaged drug product**’ class is the superclass of all of the classes that correspond with NDCs, and thus one of the primary interfaces between the dron-rxnorm module and the dron-upper module. At present, RxNorm contains insufficient information to add structure to what is essentially a flat list of NDC classes underneath packaged drug product. However, in the future, we anticipate using information from Structured Product Labels to capture things in bottles vs. boxes, for example, and adding that level of structure underneath packaged drug product.

### Modularization

**Figure 4:**
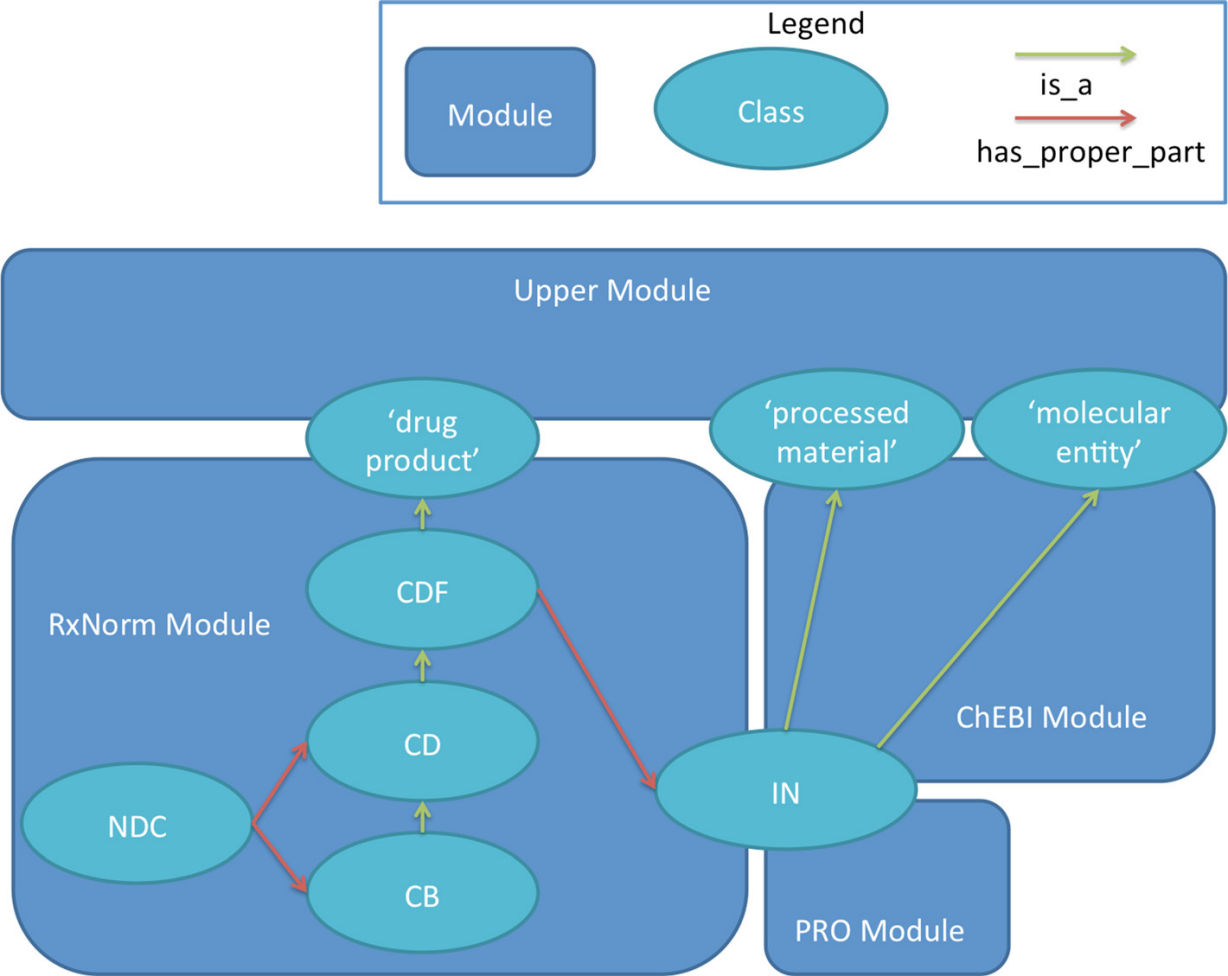
**DrOn Infrastructure.** The relationship of DrOn modules to key DrOn classes.

The ability to incorporate additional sources of information has been a key requirement for the build process. To help facilitate this ability, we developed DrOn in a modular fashion. Currently, DrOn has five different modules: **dron-full, dron-chebi, dron-rxnorm, dron-pro**, and **dron-upper**. Figure 4 illustrates the relationships of these modules to key classes in DrOn.

The **dron-full** module is simply a connector that imports the other modules. It is so named on the assumption that certain subsets of the modules may prove useful enough to warrant lighter versions of the ontology.

The **dron-chebi** module contains all of the annotations for the ingredients mapped to ChEBI (as described in the section entitled *Mapping to ChEBI*). It also contains all of the ChEBI superclasses of the ingredients and their upper level is-a structure in ChEBI.

The **dron-rxnorm** module contains all of the information mined from RxNorm, which at this point of the ontology’s development, is the bulk of DrOn’s information. It includes the NDCs, though we plan to split the NDCs from the rest of the RxNorm module in future work.

The **dron-pro** module includes everything imported from the Protein Ontology (PRO). At present, it is very small and only contains the 'protein’ and 'somatotropin’ classes from PRO. As stated above, we imported these classes to represent somatotropin as a drug ingredient, which previously was erroneously mapped to a role in ChEBI.

The **dron-upper** module contains the hand-curated upper-level ontology onto which the other modules are mapped [8].

This modularization brings two major benefits: development simplicity and increased scalability. By creating logical divisions and well-defined interfaces between the modules, we can more easily maintain each module separately without significantly affecting the other modules. Additionally, as each module grows in size, we can distribute the processing and creation of the ontologies to different servers, making it simpler to scale the process.

### Validation

We validated the design of DrOn by building a web-based software application that supports our primary, driving use case. This use case was to enable comparative-effectiveness research at the University of Arkansas for Medical Sciences, where researchers wish to study pharmacy claims datasets. To do so, they need to pull all claims where the drug product dispensed meets certain criteria. For example, author WRH was part of a research team wherein a student had to manually identify all drug products that contain acetaminophen. We built a web application that uses DrOn to support this use case; users can search for all NDCs that either contain a specific ingredient or contain an ingredient that has a specific disposition (such as beta-adrenergic receptor blockade). This web application is accessible at http://ingarden.uams.edu/ingredients.

Using this application, a user can find nearly 20,000 different packaged drug products that contain acetaminophen. Table 4 shows a number of ingredients or ingredient dispositions along with the number of NCDs the application finds associated with them by querying DrOn. In previous work [8], we used this application to test the results of a manually created list of acetaminophen NDCs against the NDCs found in DrOn. We found that DrOn contained every NDC found in the manually curated list.

**Table 4:** Several ingredients and ingredient dispositions and the number of NCDs found associated with them in DrOn

**Ingredient or disposition**	**Number of NDCs**
Acetaminophen	19,399
Ibuprofen	5,774
function-inhibiting L-type voltage-gated calcium channel binding disposition	9,650
function-inhibiting vitamin K epoxide reductase binding disposition	1,893

### Future work

Future work includes addressing limitations in the current process. One of the more egregious examples is the lack of a link from the various drug products to their dose forms (e.g., drug capsule). Nearly all of the most common dose forms are already in the upper level of the ontology (dron-upper module), but the CDFs are not properly related to them. This is due to (1) time constraints and (2) the dubious ontological nature of some of the dose forms found in RxNorm. For example, 'inhaler’ does not refer to the form of the drug but instead to its container (which also serves the role of drug delivery device). But the form of the drug itself is a solution or suspension contained in the inhaler. Note that the presentation form in this case (e.g., solution) differs from the administration form (e.g., aerosol).

Another issue is the lack of a full logical definition for some of the terms. For instance, only a small subset of the parts of each drug product is defined. A CDF has information about its dose form, its route of administration, and its active ingredients. As of the writing of this paper, only active ingredients are represented in the ontology, though dose forms are mostly represented. Even these, however, are still not fully developed, generally lacking class restrictions.

The final issue with the process is the need for manual interaction. Although each step in the process is automated, they are not tied together in a coherent way. We expect that some manual intervention will always be needed as we continue to mine updated information from these sources, but there is significant room for improvement in connecting the various segments of the overall process flow and fully automating the less ontologically nebulous steps.

Since DrOn is already large and will likely increase in size as we incorporate more sources and as more drug products are manufactured, we expect that we will run into difficulties managing generation of, and reasoning over, the ontology. One potential solution we intend to investigate is to reason over modules individually and combine the results. We also intend to create more manageable subsets of DrOn, which should allow users to work with only the portions of DrOn that they need for a particular use case.

## Competing interest

The authors have no competing interests.

## Authors’ contributions

Author EJ performed most of the historical analysis of RxNorm, including nearly all the work described under 'Mining RxNorm’. He also contributed most of the text of this section. Author WRH contributed to the historical analysis of RxNorm, guiding much of EJ’s work. He also built the dron-upper module, was involved in mapping the other modules to the proper places in the ontology, and manually curated the three dispositions not taken from ChEBI. Additionally, he helped draft the manuscript. Author MB provided ontological guidance in the construction of the ontologies and helped draft the manuscript. Author JH performed most of ETL process using the data gathered by EJ, programatically created most of the modules of the ontology, incorporated the manually-curated dispostions into the database, and helped draft the manuscript. All authors read and approved the final manuscript.
